# The dynamical signature of anhedonia in major depressive disorder: positive emotion dynamics, reactivity, and recovery

**DOI:** 10.1186/s12888-018-1983-5

**Published:** 2019-02-08

**Authors:** Vera E. Heininga, Egon Dejonckheere, Marlies Houben, Jasmien Obbels, Pascal Sienaert, Bart Leroy, Joris van Roy, Peter Kuppens

**Affiliations:** 1 Research group of Quantitative Psychology and Individual Differences, KU Leuven, Tiensestraat 102 - bus 3713, 3000 Leuven, Belgium; 2 KU Leuven, Academic Center for ECT and Neuromodulation, Leuven/Kortenberg, University Psychiatric Center KU Leuven, Leuven, Belgium; 3 Psychiatric Hospital Alexianen Tienen, Tienen, Belgium

**Keywords:** Depression, Consummatory anhedonia, Experience sampling method (ESM), Daily life, Positive emotions, Positive affect, Emotion dynamics, Pleasure loss, Reward, Mood brightening effect

## Abstract

**Background:**

Major Depressive Disorder (MDD) is the leading cause of disability worldwide. The cardinal features of MDD are depressed mood and anhedonia. Anhedonia is defined as a “markedly diminished interest or pleasure in all, or almost all, activities of the day”, and has generally been investigated on group-level using retrospective data (e.g. via questionnaire/interview). However, inferences based on group-level findings not necessarily generalize to daily life experiences within individuals.

**Methods:**

We repeatedly sampled pleasurable experiences within individuals’ daily lives by means of Experience Sampling Methods, and compared how positive affect unfolded in the daily life of healthy controls versus patients diagnosed with MDD and anhedonia. We sampled Positive Affect (PA) and reward experiences on 10 semi-random time points a day, for seven days in the daily lives of 47 MDD patients with anhedonia, and 40 controls.

**Results:**

Multilevel models showed that anhedonia was associated with low PA, but not to differences in PA dynamics, nor reward frequency in daily life. In reaction to rewards, MDD patients with anhedonia showed no difference in their increase in PA (i.e., PA reactivity), and showed no signs of a faster return to baseline thereafter (i.e., PA recovery).

**Conclusions:**

Our results suggest that the dynamical signature of anhedonia in MDD can be described best as a lower average level of PA, and “normal” in terms of PA dynamics, daily reward reactivity and reward recovery. Preregistration: https://osf.io/gmfsc/register/565fb3678c5e4a66b5582f67. Preprint: https://osf.io/cfkts

**Electronic supplementary material:**

The online version of this article (10.1186/s12888-018-1983-5) contains supplementary material, which is available to authorized users.

## Background

The word anhedonia is derived from the Greek words “An” and “hédoné”, literally meaning without pleasure. In clinical practice, anhedonia is defined as a “markedly diminished interest or pleasure in all, or almost all, activities of the day” [1], suggesting pleasure experiences in anhedonia are flat and blunted. In line with this definition, a meta-analysis showed a link between depressive symptoms and more flat or inert PA (i.e., greater spillover from one moment to the next). Yet, in a first exploration of subclinical anhedonia in daily life [2], anhedonia was associated with greater variability (i.e., greater variance in scores) and greater instability (i.e., greater mean squared successive differences between assessments) in positive emotions. The findings of greater variability and instability are remarkable, as they do not conform to the picture of being flat and blunted during almost all activities of the day. Further study of these positive emotion dynamics is essential for understanding anhedonia; particularly, how they translate to the daily life of anhedonic patients diagnosed with MDD.

Positive emotion dynamics originate from how individuals react to and recover from rewards they encounter in daily life [3]. In this respect, depression theory predicts blunted reactivity to positive events or positive contexts [4, 5], a prediction that is consistently supported by findings from laboratory studies (see for a meta-analyses: [6]). In reaction to positive stimuli or contexts in daily life, however, Ecological Momentary Assessment (EMA) studies indicate either an equal change in mood and emotions [2, 7, 8, 9], or evidence for the opposite: a slightly greater emotional change in reaction to positive stimuli or contexts (i.e., mood brightening effects; in high-arousal PA [2]; or in both PA and Negative Affect [10]).

Explanations for this discrepancy often involve methodological differences^1^, such as controlling for the cross-level interaction between psychological problems and inertia while the difference in the autocorrelations of affect (i.e., the level of inertia) between the groups is non-significant (e.g., [2]), or how reward is measured (e.g., [11]). Bakker et al. [11] have put forward that the discrepancy in findings might arise from the entanglement of events and the subjective appraisal of such, and the difference between depressed and non-depressed individuals in the nature of their selection for indicating (un)pleasant events, suggesting that behavioral engagement in rewarding activities may be a better operationalization of rewards in daily life than positive events.

Anhedonia might also manifest itself by an impaired recovery after reward reactivity [10, 12]. Koval et al. [12], for example, showed that depression was associated with a slower negative emotional recovery from negative events in daily life. The same may hold for reward recovery. In the study of Heininga et al. [2], anhedonia was associated with more positive emotional instability together with an equal reward reactivity, a combination that would arise when individuals with anhedonia would recover faster from reward. Supportive evidence for such patterns comes from Wichers, Lothmann, Simons, Nicolson, and Peeters [13], who showed that, compared to controls, the uplift in PA after physical activity was lost more rapidly in individuals diagnosed with MDD in the past. Whether the uplift in PA after positive events or rewards is also more rapidly lost in patients with anhedonia who currently fulfill a MDD diagnosis is yet to be investigated.

Taken together, the dynamical signature of anhedonia is yet to be revealed in a clinical sample. To address this gap, we largely replicate the analyses done in the subclinical study of Heininga et al. [2] and compare Positive Affective (PA) dynamics, reward reactivity, and reward recovery between controls and MDD patients with anhedonia. After data collection, but prior to accessing the data (for the full data analysis plan, please see: https://osf.io/gmfsc/register/565fb3678c5e4a66b5582f67, we hypothesized that MDD patients with anhedonia would experience:

1. Less rewards^2^;
2. Lower levels of momentary PA;
3. More variability in PA (i.e., larger affective spread);
4. More instability in PA (i.e., greater consecutive emotional change);
5. More inert PA (i.e., stronger moment-to-moment correlation);
6. Different reward reactivity (i.e., greater increase/decrease in affect after rewards);
7. Faster reward recovery (i.e., steeper slope, and less time needed to return to baseline).

## Methods

### Recruitment procedure

Clinicians screened for patients at intake in three Belgian psychiatric wards: KU Leuven hospital UPC Sint-Anna; UPC De Weg/ Onderweg; and the Broeders Alexianen

Tienen hospital ward Prisma II. Whereas patients with MDD were at the beginning of their residential treatment (i.e., inpatients), patients with BPD were at the beginning of their ambulatory treatment program (i.e., outpatients).

Admission to specialized mental health care in Belgium is provided via different routes, and does not necessarily involve an acute risk of suicide or life-threatening self-neglect due to depression. The admission can be direct, via a referral from a medical doctor or psychiatrist, or indirect, after hospitalization. When hospitalized (e.g., due to acute risk of suicide), patients are first admitted to one of the Psychiatric Departments of the General Hospitals. These are department of a general hospital where care is provided to psychiatric patients which distinguish themselves from specialized psychiatric hospitals or other treatment institutions. From here, patients are transferred to units that provide specialized mental health care.

If a patient was judged eligible for enrollment in the study, a clinically trained researcher interviewed the patient using the Dutch version of the Structured Clinical Interview for DSM axis-IV Axis I disorders (SCID-I) and the Borderline Personality Disorder (BPD) subscale of the DSM axis-II disorders (SCID-II). Patients were included if they met the criteria for one of the mood disorders, and excluded if they were acutely psychotic; acutely manic; addicted; or diagnosed with a (neuro-)cognitive disorder.

The sample of the healthy controls was matched on age and gender with the group of clinical patients, and recruited via advertisements, social media, flyers, and by the Experiment Management System of the KU Leuven university. Exclusion criteria were current illness.

After enrollment, participants were invited to the lab or visited in the psychiatric hospital. After the nature of the procedures had been fully explained, informed consent of participants was obtained. Next, participants were asked to perform a computer task and filled out baseline questionnaires. The next day, after receiving two trial EMA-assessments between 6.30 PM and 8.00 PM, the EMA-part of the study started.

### EMA assessments

EMA assessments were semi-randomly presented on a Motorola Defy Plus smartphone device, using a custom made EMA software program MobileQ (Meers, K., Dejonckheere, E., Kalokerinos, E., Rummens, K. and Kuppens, P.: MobileQ, in preparation), ten times a day between 9.30 am and 9.30 pm, for seven days, within equal intervals per day of 66 minutes (i.e., maximum 70 assessments per person). The total number of questions asked per assessment was 27 (eight on emotions; one on social expectancies; four on emotion regulation; five on context; nine on psychiatric symptoms). Questions were clustered, the clusters were administered randomly, and questions within cluster random as well. No reminders were sent, and participants were not contacted if they missed assessments. Participants received e35,- for a compliance rate above 75%, and e5,- less for every 10% lower.

### Data exclusions and subsample selection

The participants of this study consisted of 47 patients (10 outpatients and 37 inpatients) diagnosed with anhedonia and MDD (and possibly other psychiatric diagnosis; please see comorbidity rates), and 40 people without psychological complaints (i.e., healthy controls). This is a subset drawn from a larger study on emotion dynamics in people with Major Depressive Disorder, Borderline Personality Disorder, Bipolar Disorder, and people without psychological complaints.

From the initially 90 patients enrolled, three quitted during the baseline assessments. Two patients had to be excluded because they had malfunctioning devices during the study; and seven others due had a <50% compliance rate. Of the remaining 78 patients, 38 patients were diagnosed with MDD (and possibly other psychiatric diagnoses but not BPD), 20 diagnosed with BPD (and possibly other psychiatric diagnoses but not MDD), and 20 diagnosed with both MDD and BPD. The 20 patients who were diagnosed with BPD but not MDD were excluded from the analyses, leaving 58 patients diagnosed with MDD (of which 20 were also diagnosed with BPD). Of these 58 MDD patients, five were excluded because of past (hypo) manic episodes^3^, leaving 53 eligible patients. Of these 53 patients, 47 were diagnosed with anhedonia during the SCID interview (89%).

Anhedonia was assessed during the SCID interview using following questions: “Did you lose interest or pleasure in things you usually enjoyed? (What was that like?)” and, if yes, “When was that? Was that nearly every day? How long did it last? As long as two weeks?”. Based on the participants’ answers, anhedonia was rated “absent”, “subthreshold”, or “present”. Patients were included in the subsample if anhedonia was rated “present”. The Structured Clinical Interview for DSM axis-I disorders (SCID-I), is an extensive. semi-structured diagnostic interview designed to determine the presence of symptoms for a range of disorders, including depression (First, Spitzer, Gibbon, & Williams, [14]; Gibbon, Spitzer, Williams, Benjamin, & First [15]). The SCID was administered by a trained clinician and, based on a random sample of seven audio recordings of these clinical interviews, a second independent trained clinician rated symptoms and diagnoses of a random subsample of seven interviews of patients and healthy controls. The interrater reliability between the two raters was Cohen’s *κ* = .93 on diagnostic level and Cohen’s *κ* =.92 on symptom level. For our analyses, and as preregistrated, we transformed/collapsed the three answer categories into a dichotomous variable that reflected if anhedonia was present (i.e., rated as “present”) or not (i.e., “absent” or “subthreshold”).”

From the 44 controls that were initially enrolled, one participant was excluded because of a compliance rate <50%, and three because they met the SCID criteria for a current psychiatric disorder. The final subsample consisted of 40 controls, and MDD patients with anhedonia.

### Measures

#### Positive affect (PA)

PA was conceptually based on the extended Positive and Negative Affect Schedule (PANAS; Watson, Clark, & Tellegen, [16]). Instead of all the ten original items, to prevent participants from being overburdened with questions when sampling them 10 times a day, we chose those the three items with which we could cover the complete affect grid (Russell & Barrett, [17]): “How euphoric do you feel at the moment?” (high arousal); “How happy do you feel at the moment?” (neutral arousal); and “How relaxed do you feel at the moment?” (low arousal). Participants answered on a sliding scale ranging from “not at all” on the left (0) to respectively “very euphoric/happy/relaxed” on the right (100).

Cronbach’s alpha was .03 within-subject^4^, and .99 between subject.

#### Psychological rewards

Reflected (1) if participant answered “Yes” when asked “Did something positive happen since the last assessment?”, and (0) if the answer was “no”.

#### Behavioral rewards

Reflected (1) if the participant indicated to be engaged in “sport” or “hobby”, when asked “What kind of activity were you doing at the moment?”, or “friends” or “partner” when asked “Who are you with at the moment?”. Reflected (0) if the participant did not engage in such potentially rewarding behavior.

### Statistical procedures

We investigated our hypotheses by logistic regressions and cross-lagged multilevel models in R [18], and Rmarkdown [19, 20, 21, 22, 23, 24, 25, 26, 27–29]. For more information and equations, please see the Additional file 1.

## Results and discussion

### Descriptives

The sample consisted of 41% men. Mean age was of 36.79 (for demographic statistics by group, please see Table 1). MDD patients with anhedonia did not differ from controls in mean age ∆*M* = *−*2*.*90, 95% CI [*−*8*.*16, 2*.*36], *t*(84*.*90) = *−*1*.*10, *p* = *.*275, nor in the proportion of men ∆*M* = 0*.*02, 95% CI [*−* 0*.*19, 0*.*23], *t*(82*.*54) = 0*.*19, *p* = *.*847. On average, the EMA assessments were spaced apart 134.50 minutes, and took approximately 2’2” (*SD* = 37”) to be filled out. Compliance rate was 89% and, compared to controls, the anhedonic group filled out fewer assessments (∆*M* = 0*.*06, 95% CI [0*.*02, 0*.*10], *t*(82*.*19) = 3*.*25, *p* = *.*002). For more information on the kurtosis and skewness of variables, please see the Additional file 1.

**Table 1:** Demographics in control groups and anhedonia MDD group

	Control group				Anhedonia MDD group			
	Mean	SD	Min	Max	Mean	SD	Min	Max
Men	0.42	0.50	0.00	1.00	0.40	0.50	0.00	1.00
Age	35.23	11.54	21.00	64.00	38.13	13.12	18.00	61.00
Compliance	0.92	0.07	0.70	1.00	0.86	0.11	0.54	0.99
Time	134.42	0.58	133.15	135.78	134.57	0.45	133.77	135.53
PastMDE	0.02	0.16	0.00	1.00	0.72	0.45	0.00	1.00

‘Men’; ‘Compliance’; and ‘PastMDE’ are proportions. ‘PastMDE’ reflects the proportion of participants who had a Major Depressive Episodes in the past. ‘Time’ is the time passed since the last assessment is in minutes

#### Comorbidity

In addition to current MDD and current anhedonia, approximately 72% of the patients from the anhedonic group had experienced a Major Depressive Episode in the past, and 53% were diagnosed with another disorder. The top three of current comorbid disorders were:

1. Borderline personality disorder (32%);
2. Generalized anxiety disorder (13%);
3. Social phobia (9%); Panic disorder with agoraphobia (9%)

## Results and discussion

### H1: Less rewards

Logistic regression results on the aggregated within-subject reward frequencies indicated that the anhedonic group did not experience a lower frequency of Psychological Rewards than the control group (*B* = -0.01; *z* = -0.03; *p* = 0.487), nor a lower frequency of Behavioral Rewards (*B* = -0.12; *z* = -0.60; *p* = 0.275)^5^. Finding no differences in reward frequencies between the te groups is different from what most other EMA studies have shown. That is, compared to controls, individuals with minor depression or MDD typically show a lower frequency of positive events ([7, 10]; but see: [9]). This lower frequency was also found in anhedonic boys around 14 years of age [8], and anhedonic adult women between 18 and 24 years old [8].

### H2: Lower level of PA

In line with our hypothesis, the random intercepts multilevel model showed that indeed MDD patients with anhedonia experienced lower levels of PA than controls (*t(84.96)* = -7.72, *p* < 0.001). Although of larger magnitude, this difference is in line with the subclinical study into anhedonia [2].

### H3: More variability in PA

Results from general linear modelling showed that MDD patients did not have a greater variance in PA than controls ∆*M* = 18*.*27, 95% CI [*−*30*.*01, 66*.*55], *t*(80*.*58) = 0*.*75, *p* = *.*454. In the subclinical EMA study into anhedonia by Heininga et al. [2], however, the PA variance was found larger in individuals with anhedonia. The lack of difference in variance between the groups is remarkable, as typically also other forms of compromised well-being are related to a greater dispersion of PA scores [30].

### H4: More instability in PA

The random intercept multilevel model showed that there was not more instability in PA in MDD patients with anhedonia than in controls (*B* =-0.18; *t(85.42)* = -1.24; *p* = 0.110).

Previously, anhedonia was associated with a higher Mean Successive Squared Difference (MSSD). The MSSD has been proposed as a more valid and more reliable measure of the within-subject affective lability than the variance [31], and in the subclinical study into anhedonia of Heininga et al. [2] the MSSD was larger in subjects with anhedonia than without.

### H5: More inert PA

As shown in Table 2, random effect multilevel models showed that MDD patients with anhedonia did not have more inert PA than controls (*B* = -0.01; *t(4349)* = -0.49; *p* = 0.312).

**Table 2:** Inertia in PA, and PA reactivity to Reward (full model)

	Psychological Reward					Behavioral Reward				
	Estimate	Std. Error	df	t value	Pr(>|t|)	Estimate	Std. Error	df	t value	Pr(>|t|)
(Intercept)	43.01	1.71	85.04	25.17	0.00	43.08	1.79	84.61	24.10	0.00
LaggedPA	0.32	0.02	4333.62	17.04	0.00	0.31	0.02	4360.64	15.89	0.00
Anhedonia	−19.78	2.33	85.39	−8.50	0.00	−18.19	2.43	84.98	−7.47	0.00
Reward	6.26	1.04	53.75	6.03	0.00	3.74	0.74	72.38	5.02	0.00
LaggedPA:Anhedonia	−0.01	0.03	4349.30	− 0.49	0.62	0.03	0.03	4354.67	1.16	0.25
Anhedonia:Reward	3.65	1.44	57.15	2.53	0.01	−1.50	1.05	76.44	−1.44	0.16

Dependent variable is Positive Affect (PA); PA is the average of feeling relaxed, happy, and euphoric; LaggedPA is the person-mean centered lagged variable of PA (i.e., PA on t-1);BR stands for Behavioral Reward

At first glance, this findings seems remarkable, as more inert PA seems implied in the clinical and theoretical concept of depression (e.g., having a “flattened” emotional landscape). In a large EMA meta-analysis, depressive symptoms were linked to greater variability and instability in PA, but also to more inert PA [30]. However, the previous subclinical EMA study into anhedonia did not find a difference in PA inertia. Although the authors concluded that a six hour time frame was a too large time frame to detect PA spill-over effects in anhedonia, our results using a EMA design with approximately 70 minutes in between suggests that that is not the case.

### H6: Different PA reactivity to rewards

**Fig. 1:**
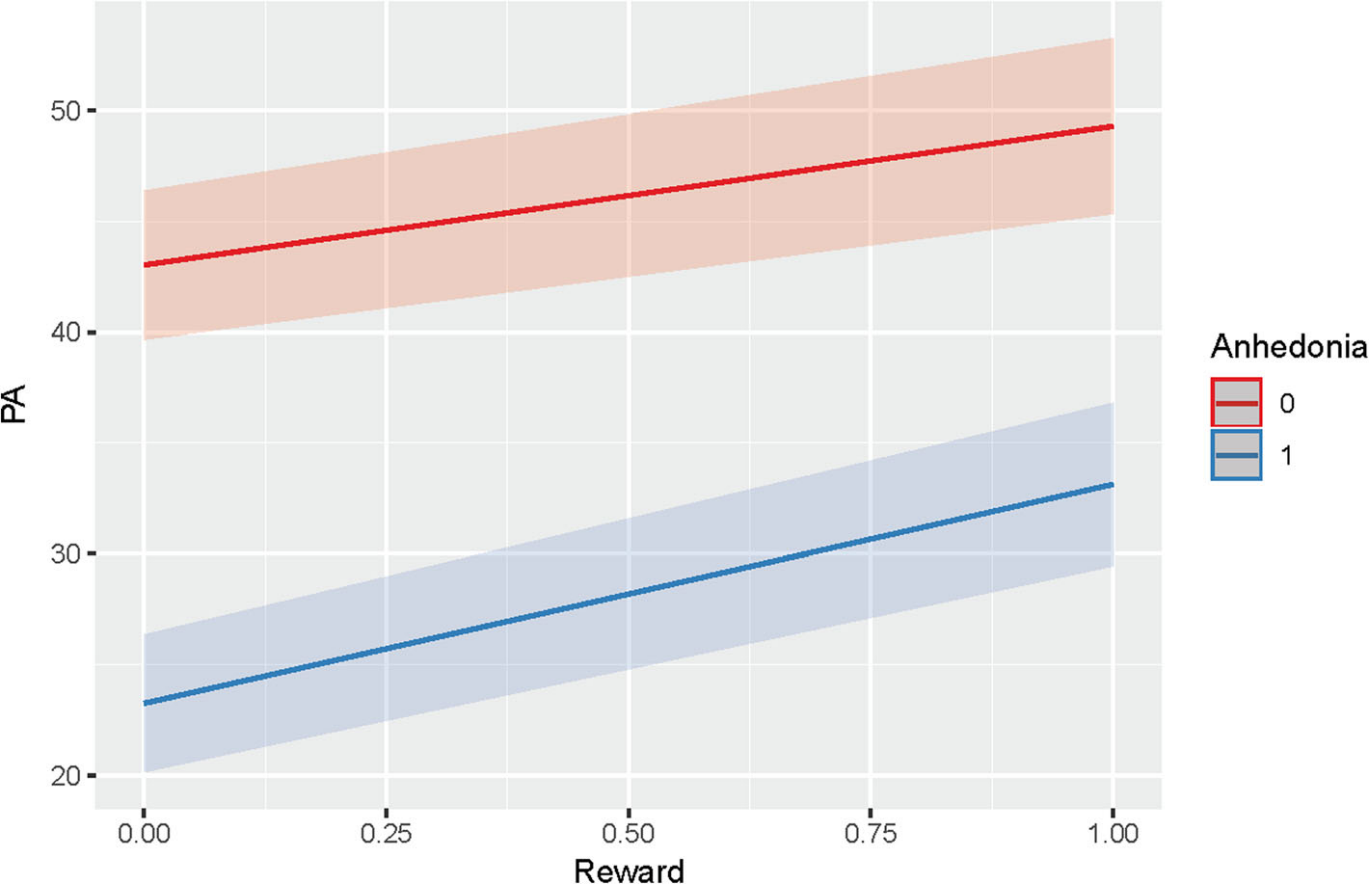
PA reactivity to rewards (trimmed model), with a steeper positive slope reflecting a greater increase in PA after a reward

As shown in Tables 2 and 3, MDD patients with anhedonia showed a difference in the amount of change in PA after Behavioral Rewards at *p* <.05 but not *p* <.01. Given that the autocorrelation did not differ between groups (i.e., PA inertia), we reran the analyses after omitting this part of the model (for the statistical formula, please see Additional file 1). Results of this more parsimonious or trimmed model were in the same direction as in the original or “full” model: considering the family-wise VeffLi-Bonferroni-corrected alpha level of *p* <.01, MDD patients with anhedonia showed no difference in increase in PA in reaction to Psychological Rewards (*B* = 3.65; *t(57)* = 2.53; *p* = 0.014; see Fig. 1), nor to Behavioral Rewards (*B* = − 1.50; *t(76)* = − 1.44; *p* = 0.155).

**Table 3:** Reactivity in PA to rewards (trimmed model)

	Psychological Reward					Behavioral Reward				
	Estimate	Std. Error	df	t value	Pr(>|t|)	Estimate	Std. Error	df	t value	Pr(>|t|)
(Intercept)	43.01	1.71	85.04	25.16	0.00	43.09	1.79	84.62	24.12	0.00
LaggedPA	0.32	0.01	4348.66	22.89	0.00	0.33	0.01	4355.76	22.98	0.00
Reward	6.27	1.04	53.73	6.04	0.00	3.70	0.74	71.94	5.00	0.00
Anhedonia	−19.77	2.33	85.38	−8.49	0.00	−18.21	2.43	84.97	−7.48	0.00
Reward:Anhedonia	3.61	1.44	56.80	2.51	0.01	−1.43	1.04	75.71	−1.38	0.17

Dependent variable is Positive Affect (PA); PA is the average of feeling relaxed, happy, and euphoric; LaggedPA is the person-mean centered lagged variable of PA (i.e., PA on t-1); BR stands for Behavioral Reward. To maintain a familywise error rate of .05 over all analyses of PA reactivity (see Additional file 1), a Bonferroni-correction of a’ = 1-(1-a)1VeffLi was be applied with VeffLi being the ‘effective number’ of independent tests corrected for the correlation amongst the different predictors. Using the approach proposed by Li & Ji [39], we calculated that a *p* < .01 is required to keep Type I Error Rate at 5% accordingly (for the full calculations, please see the Additional file 1 or Rmarkdown file of the Additional file 1 on Open Science Framework: https://osf.io/8gxrw/)

#### Different NA reactivity to rewards

Given that we preregistered that if find we find a greater PA reactivity in MDD patients with anhedonia than in controls (at *p*<.05), we also investigated whether these effects were present with regard to Negative Affect (NA^6^) as greater reward reactivity in both PA and NA would suggest a mood brightening effect in MDD anhedonia.

After substituting PA by NA in the “full” model (see Equation 4a in the Additional file 1) showed that the autocorrelation of NA did not differ by group. The “trimmed” model showed that the mood of MDD patients with anhedonia “brightened” more in response to a Psychological Reward than the mood of controls (*B* = 3.61; *t(57)* = 2.51; *p* = 0.015), but the p-value of the effect does not survive the VeffLi-Bonferroni-correction to maintain the 5% family-wise error rate. In previous EMA-research, the mood brightening effect has been reported by at least two EMA studies so far (only in NA by [7]; only in high-arousal PA by [2]; and in both PA and NA by [10]), and the difference in findings may (partly) reside in the methodological differences, as well as (partly) in the operationalization of PA and NA.

With regard to PA reactivity, we found no differences. This finding is in line with that of Bakker et al. [11], who also found no moderating effects of depressive symptoms on the association between active behavior and PA. However, Bakker et al. [11] argued that this finding is in line with the mounting laboratory and neuroscientific studies that suggest depression affects anticipatory rather than the consummatory reward reactivity. One EMA study reported that MDD was associated with blunted reward responses in both the consummatory and anticipatory aspect of reward experience [32], but operationalized consummatory reward response not as the increase in PA in response to reward but as a lower rating of pleasurableness of potentially rewarding behaviors (i.e., defined here as the intensity of reward).The different operationalization of reward and reward reactivity thus lead to different conclusions.

Bakker et al. [11] have put forward that behavioral engagement in rewarding activities are a better operationalization of rewards in daily life than positive events, because the subjective appraisal of a positive event rating is inextricably intertwined with PA. High PA could be the very reason that participants rate an event as positive. That we found the counterintuitive “mood brightening effects” with regards to Psychological Rewards but not Behavioral Rewards might attest this notion, although we cannot exclude that this difference arose from a suboptimal operationalization of Behavioral Rewards. Whereas Psychological Rewards were events that were appraised as positive by the participant, we defined Behavioral Rewards as behaviors of which we thought they would be rewarding in general, such as engaging in hobbies and being together with one’s partner. However, engagement in one’s hobby does not always have to be rewarding, and neither does the company of one’s partner. It may thus well be that we did not find the counterintuitive “mood brightening effects” with regard to Behavioral Rewards because our measure was too general or imprecise.

Given that different operationalization’s of reward and reward response in daily life seem to result in inconsistencies and may thus hamper the accumulation of new knowledge, we call for more research into the best way to measure “reward” and “reward response” in daily by means of EMA. While the “best” operationalization remains unclear, EMA researchers can add to current knowledge by adding extra checks for event-selection effects to their analysis plan. For example, to make outcomes more comparable across differently designed EMA studies, researchers who used a design in which participants always had to report a negative or positive event score could include checks on whether their results would differ after dichotomizing their reward-variable (e.g., zero reflecting the bottom 25% of all event ratings, and one reflecting the top 25% ratings). That way, the outcomes on reward response (e.g., PA reactivity) may be better comparable to those outcomes based on EMA studies in which participants could chose to report an event or not. Researchers who investigate PA reactivity and find no differences between depressed and non-depressed individuals’ inertia could report on their results both when using the “full model” (i.e., including the cross-level interaction with inertia) and when using the “trimmed model” (i.e., excluding the cross-level interaction with inertia; see also [2]).

### H7: Faster PA recovery

Faster recovery was operationalized in terms of slope and duration.

#### Steeper slope

**Fig. 2:**
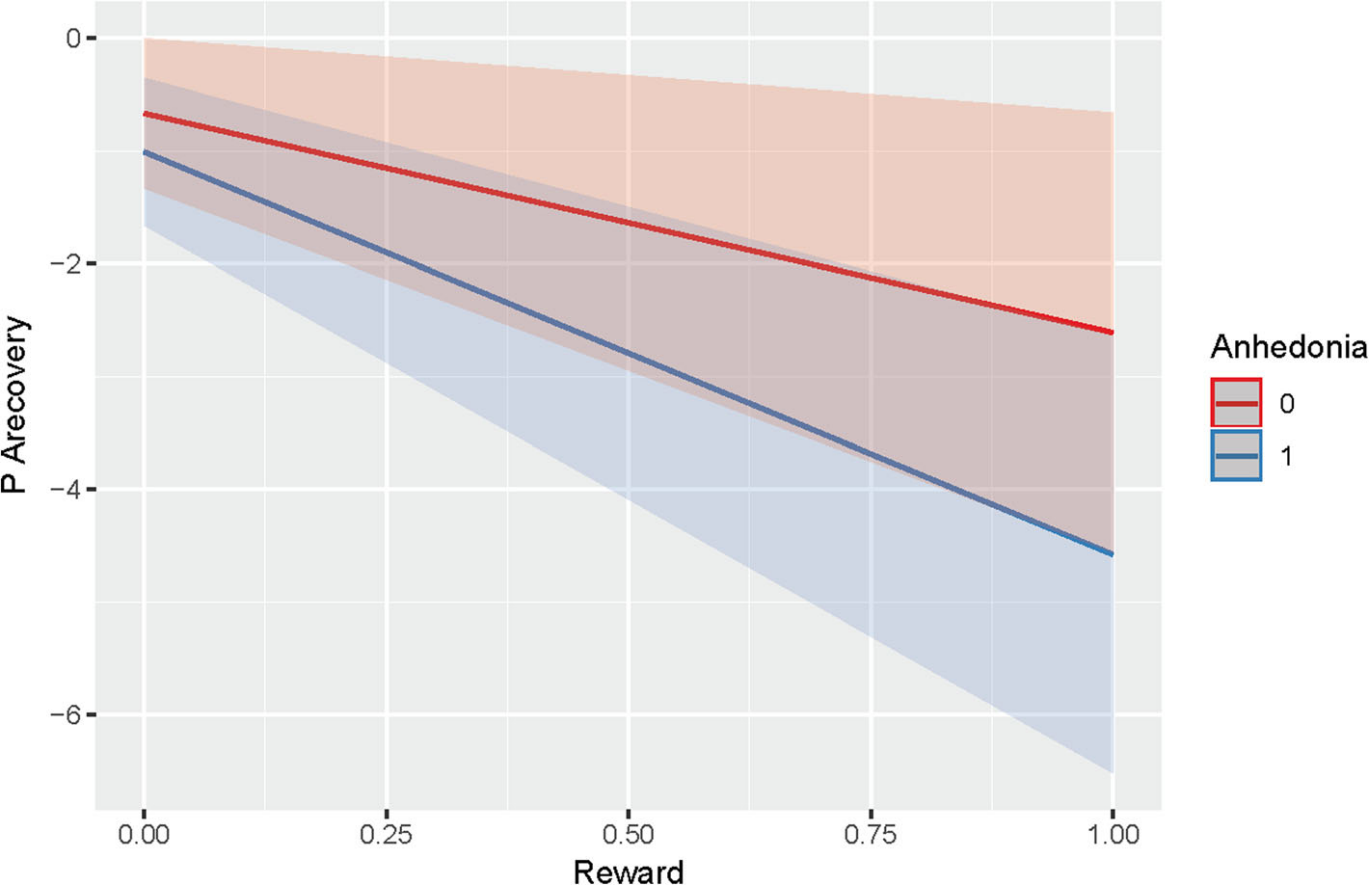
PA recovery from reward, with a steeper negative slope reflecting a faster recovery in PA after a reward

Table 4 shows that MDD patients with anhedonia did not recover faster after experiencing Psychological Rewards (*B* = -1.63; *t(68)* = -1.10; *p* = 0.137; see also Fig. 2), nor after Behavioral Rewards (*B* = 0.33; *t(78)* = 0.23; *p* = 0.410)^7^.

**Table 4:** PA Recovery from rewards (steepness of slope)

	Psychological Reward					Behavioral Reward				
	Estimate	Std. Error	df	t value	Pr(>|t|)	Estimate	Std. Error	df	t value	Pr(>|t|)
(Intercept)	− 0.12	0.37	3025.16	− 0.34	0.73	− 0.21	0.38	2740.91	− 0.57	0.57
Reward	−1.95	1.05	58.53	−1.85	0.07	−1.30	1.07	82.70	−1.22	0.23
Anhedonia	−0.34	0.47	3016.11	−0.72	0.47	0.43	0.50	2717.76	0.86	0.39
PAreactivity	−0.42	0.02	3079.97	−26.37	0.00	−0.46	0.02	2790.69	−27.44	0.00
Time2	0.00	0.00	3065.95	4.08	0.00	0.00	0.00	2762.56	4.02	0.00
Reward:Anhedonia	−1.63	1.48	68.01	−1.10	0.27	0.33	1.46	77.50	0.23	0.82

Dependent variable is PA recovery difference score (PA t + 1 - PA) provided that a Psychological Reward has not been reported on t + 1 (again); PA = the average of feeling relaxed, happy, and euphoric; Time = the number of minutes between t-1 and t; Reward = Psychological Reward, asked as ‘Did you experience a positive event since the last assessment?’ with possible answers Yes (1) or no (0); Anhedonia = participant in control group (0) or group of participants with MDD and anhedonia (1)?; PA reactivity = person-mean centered PA reactivity difference score (i.e., PA - PA t-1). To maintain a familywise error rate of .05 over all analyses of PA recovery (see Additional file 1), a Bonferroni-correction of a’ = 1-(1-a)1VeffLi was be applied with VeffLi being the ‘effective number’ of independent tests corrected for the correlation amongst the different predictors. Using the approach proposed by Li & Ji [39], we calculated that a *p* < .01 is required to keep Type I Error Rate at 5% accordingly (for the full calculations, please see the Additional file 1 or Rmarkdown file of the Additional file 1 on Open Science Framework: https://osf.io/8gxrw/)

#### Shorter duration

As shown in Table 5, the average minutes to return to baseline (i.e., the level of PA at *i-1* ) after experiencing Psychological Rewards were 87.93 minutes, and for Behavioral Rewards 96.66 minutes. Patients with MDD and anhedonia did not show a faster recovery after Psychological Rewards in terms of minutes needed to return to baseline (*B* = -4.27;*t*(262) = -0.65; *p* = 0.259), nor after Behavioral Rewards (*B* = 8.19; *t*(245) = 1.03; *p* =0.152).

**Table 5:** PA recovery from rewards (duration in minutes)

	Psychological Reward					Behavioral Reward				
	Estimate	Std. Error	df	t value	Pr(>|t|)	Estimate	Std. Error	df	t value	Pr(>|t|)
(Intercept)	87.93	4.45	262.00	19.78	0.00	96.66	6.09	245.00	15.87	0.00
PAreactivity	0.30	0.25	262.00	1.18	0.24	1.18	0.34	245.00	3.52	0.00
Anhedonia	−4.27	6.59	262.00	−0.65	0.52	8.19	7.96	245.00	1.03	0.30

Dependent variable is the number of minutes needed to recover PA (i.e., come back to baseline) after experiencing a reward;PAreactivity is the person-mean centered amount of increase in PA on time point t in comparison to t-1 (i.e., PA reactivity, but now modelled as a difference score: PA minus PA on t-1); Reward refers to Psychological Reward experienced somewhere between t-1 and t

Across approximately six-hour time frame, the previous subclinical study found increased variance and MSSD high-arousal PA in combination with a greater increase in high-arousal PA, leading them to conclude that anhedonia may be associated with a normal to greater increase or “spike” in PA in reaction to a reward followed by a sharp decrease or “crash” in PA thereafter [2]. Our results do not support this speculative conclusion as anhedonia in MDD is not associated with a steeper PA recovery slope after reward, nor a shorter duration of PA recovery after reward. We showcased three different ways to statistically model PA recovery slope (see Additional file 1), largely based on what was done previously on NA [12], but there may be other alternatives. In a similar vein, there may also be alternatives to model PA recovery duration. For example, one could code the *i* on which a positive event took place 0, and make the following *i*’s reflect the number of minutes since then. Subsequently, in a multilevel analysis, this variable can be used as predictor of affect, and tests whether this effect differs between groups (Vaessen, T., Viechtbauer, W., Reininghaus, U., MERGE, Claes, S., & Myin-Germeys, I: Recovery from daily-life stressors in early and chronic psychosis, in preparation; [13, 33]). Never used on EMA data yet, but potentially promising, would be the use of cox regression models with mixed effects, or frailty models, for the analysis of clustered survival data [34–36].

Many studies have measured positive affect before, but there has not yet been much focus on validation or standardization of PA items used. Whereas many previous EMA studies used the PA items “feeling relaxed” and “feeling happy”, we were the first to use “feeling euphoric”. Our exploratory analyses revealed that the euphoria item did not change our conclusions with regard to the hypotheses. It did, nevertheless, negatively affect the Cronbach’s alpha and overall mean level of the PA scale. Future researchers are therefore advised to substitute the euphoria item by another high-arousal PA item such as feeling joyful, determined, lively, enthusiastic, cheerful, or energetic [2, 13, 37, 38].

Although speculative, and to conclude our findings on a more general level, the lower level of PA that we found in individuals with MDD anhedonia might be a dysregulation of an otherwise adaptive mechanism. According to evolutionary theories, feeling anhedonic might be an adaptive mechanism to cope with the threat of social exclusion that is dysregulated in those diagnosed with a mood disorder. Empirical research shows evidence in support of this theory, as social support is often pinpointed as an important factor in risk and resilience for the development of mood disorders, and in the recovery thereof. It would be promising to further investigate the association between anhedonia and social stress in future research.

### Study limitations

This study had several limitations. First, nearly all MDD patients also met the anhedonia criteria. Because we were unable to compare MDD patients with anhedonia to MDD patients without anhedonia, we cannot exclude that the differences we found are not specific to anhedonia, but to MDD in general.

Second, our control participants filled out their assessments at home or outside of their home, whereas 37 of the 47 patients were admitted to a psychiatric hospital environment. This difference in context might have affected the opportunities for reward, and/or the reported level of affect. That we found no difference in the proportion of rewards experienced between patients and controls on a group level, suggests that rewards might have been imposed by elements of the clinical setting or treatment (e.g., imposed by medication or medical staff). We might have found a difference if we would have sampled in the life of the patients before they started their treatment, especially for inpatients. Despite that we also had 10 outpatients in our sample, the imposed rewards in the inpatient setting might have obscured the strength of the association between MDD anhedonia and reward functioning.

Third, the recovery duration analyses were conditional on the experience of a reward and no “new” reward in the five assessments thereafter. Given that participants reported a reward in 15.22% of their total assessments (see 2), and PA recovery was conditional on rewards, there might have been small differences in recovery effects but we were unable to detect it.

Fourth, because the extended Positive and Negative Affect Schedules (PANAS; Watson & Clark, [16]) are too long to assess in daily life, and there are no validated EMA-scales for PA and NA (yet), we selected our own set of items. Given that the PANAS is highly criticized because of its overrepresentation on high-arousal and underrepresentation of low-arousal items, we choose a low-, moderate-, and high-arousal PANAS items to cover the whole affect grid (Russell & Barrett, [17]). However, in hindsight, “euphoria” might have been a suboptimal choice as this feeling appeared to be less commonly experienced and negatively affected the PA scale’s Chronbachs alpha. However, we reran our analyses after omitting the euphoria item from our scale, and results did not change (see Additional file 1).

Fifth, the average time between assessments of approximately an hour, and we cannot exclude that reward reactivity or recovery has taken place within this time frame and in between measurements.

## Conclusions

The signature of anhedonia in daily life in individuals who currently fulfill a MDD diagnosis can be predominantly described as a significantly lower average level of PA. The signature is not distinctive in its frequency of reward experience, the dynamics in PA in terms of spread, shifts, and autocorrelation, nor in its temporary increase after rewards or its recovery, at least not when using an EMA design with 10 semi-random assessments a day.

## Additional file

Additional file 1: Anhedonia in MDD – supplementary analyses.pdf. The dynamical signature of anhedonia in Major Depressive Disorder: Positive emotion dynamics, reactivity, and recovery’. The datasets used for these analyses are: 1) MyData supplement agg Heininga 2018 07 26_ANONYM.sav, and 2) MyData supplement Heininga 2018 07 26_ANONYM.sav. These Additional file 1 include: Statistical procedures, including deviations from what was preregistered (https://osf.io/4bkad/). (PDF 665 kb)

## Footnotes

1 In research devoted to the NA reactivity to stressful events in MDD, for example, EMA studies either find a blunted or mood brightening effects, depending on the question-design for events. In this strand of research, blunted responses to negative events are found when participants were asked to select their events, and had to choose between whether they had experienced a negative event or not. Mood brightening effects, to the contrary, are reported by studies that used EMA designs in which participants were asked to always report a score for the negative or positive event (e.g., think of the most stressful event, how stressful was this?).

2 The inclusion of behavioral rewards in the first hypothesis is a deviation from what was preregistrated, which read “Lower frequency of positive events (directional)” and originally thus not include the behavioral alternative operationalization of reward.

3 Please note that this is a deviation from what was preregistered, as it was initally left unnoticed that 5 MDD patients had also reported (hypo) manic episodes by which they qualify for Bipolar Disorder instead of MDD. To be able to still investigate anhedonia MDD (and possibly BPD, but not Bipolar Disorder) we decided to deviate from our preregistration and remove these participants.

4 After omitting the euphoria item, the Cronbach’s alpha of PA composed of happy and relaxed became .44 within-subject, and .99 between subject. We therefore reran the analyses after omitting the euphoria item from the PA scale. Results were similar. For more details, please see the Additional file 1.

5 The inclusion of behavioral rewards in the first hypothesis is a deviation from what was preregistrated, as in the original analysis plan only “the sixth and seventh hypothesis [would] be tested again, but now [using the behavioral] operationalization of the reward concept”.

6 The NA variable was the mean of the EMA items “How stressed do you feel at the moment?”; “How depressed do you feel at the moment”; “How anxious do you feel at the moment?”; and “How angry do you feel at the moment”, all measured on a sliding scale from 0 “not at all” to 100 “very”. Cronbach’s alpha reliability was .31 within-subject, and .996 between subject.

7 Please see an exploration of the robustness of this finding across different alternative ways of modelling PA recovery in our Supplementary Material. In these models, instead of calculating PA Recovery as a difference score, we modelled PA recovery from reward in a similar way as we modelled PA reactivity to reward. These results did not change our main conclusion after Psychological Rewards in terms of minutes needed to return to baseline (*B* = -4.27; *t(262)* = -0.65; *p* = 0.259), nor after Behavioral Rewards (*B* = 8.19; *t(245)* = 1.03; *p* = 0.152).

## References

[1] American Psychiatric Association, T. F. on D.-I (2009). Diagnostic and statistical manual of mental disorders: DSM-IV.

[2] Heininga VE, van Roekel E, Ahles J, Oldehinkel A, Mezulis A. Positive affective functioning in Anhedonic individuals’ daily life: anything but flat and blunted. J Affect Disord. 2017. 10.1016/j.jad.2017.04.029. 10.1016/j.jad.2017.04.029 28531841

[3] Kuppens P, Verduyn P (2017). Emotion dynamics. Curr Opin Psychol.

[4] Rottenberg J (2005). Mood and emotion in major depression. Curr Dir Psychol Sci.

[5] Rottenberg J, Gross JJ, Gotlib IH (2005). Emotion context insensitivity in major depressive disorder. J Abnorm Psychol.

[6] Bylsma LM, Morris BH, Rottenberg J (2008). A meta-analysis of emotional reactivity in major depressive disorder. Clin Psychol Rev.

[7] Bylsma LM, Rottenberg J (2011). Uncovering the dynamics of emotion regulation and dysfunction in daily life with ecological momentary assessment. Emotion regulation and well-being.

[8] van Roekel E, Bennik EC, Bastiaansen JA, Verhagen M, Ormel J, Engels RCME, Oldehinkel AJ. Depressive symptoms and the experience of pleasure in daily life: an exploration of associations in early and late adolescence. J Abnorm Child Psychol. 2015. 10.1007/s10802-015-0090-z. 10.1007/s10802-015-0090-z PMC4893355 26496738

[9] Thompson RJ, Mata J, Jaeggi SM, Buschkuehl M, Jonides J, Gotlib IH (2012). The everyday emotional experience of adults with major depressive disorder: examining emotional instability, inertia, and reactivity. J Abnorm Psychol.

[10] Peeters F, Nicolson NA, Berkhof J, Delespaul P, De Vries M (2003). Effects of daily events on mood states in major depressive disorder. J Abnorm Psychol.

[11] Bakker JM, Goossens L, Lange I, Michielse S, Schruers K, Lieverse R (2017). Real-life validation of reduced reward processing in emerging adults with depressive symptoms. J Abnorm Psychol.

[12] Koval P, Brose A, Pe M, Houben M, Erbas Y, Champagne D, Kuppens P. Emotional inertia and external events: the roles of exposure, reactivity, and recovery. Emotion. 2015. 10.1037/emo0000059. 10.1037/emo0000059 25844974

[13] Wichers M, Lothmann C, Simons CJP, Nicolson NA, Peeters F (2012). The dynamic interplay between negative and positive emotions in daily life predicts response to treatment in depression: a momentary assessment study. Br J Clin Psychol.

[14] Spitzer RL, Gibbon ME, Skodol AE, Williams JB, First MB. DSM-IV-TR casebook: A learning companion to the diagnostic and statistical manual of mental disorders, text rev. American Psychiatric Publishing, Inc.; 2002.

[15] Gibbon M, Spitzer RL, Williams JB, Benjamin LS, First MB. Structured clinical interview for DSM-IV axis II personality disorders (SCID-II). Am Psych Pub. 1997.

[16] Watson D, Clark LA, Tellegen A (1988). Development and validation of brief measures of positive and negative affect: the PANAS scales. J Pers Soc Psychol.

[17] Russell JA, Barrett LF (1999). Core affect, prototypical emotional episodes, and other things called emotion: dissecting the elephant. J Pers Soc Psychol.

[18] R Core Team (2013). R: a language and environment for statistical computing.

[19] Comtois D (2018). Summarytools: Tools to quickly and neatly summarize data.

[20] Elff M (2017). Memisc: Management of survey data and presentation of analysis results.

[21] Komsta L, Novomestky F (2015). Moments: Moments, cumulants, skewness, kurtosis and related tests.

[22] Kuznetsova A, Brockhoff PB, Christensen RHB (2017). lmerTest package: tests in linear mixed effects models. J Stat Softw.

[23] Lüdecke D (2018). SjPlot: data visualization for statistics in social science.

[24] Patil I (2018). Ggstatsplot: ‘Ggplot2’ based plots with statistical details.

[25] Phillips N (2017). Yarrr: A companion to the e-book “yarrr!: The pirate’s guide to r”.

[26] Core Team R (2017). Foreign: read data stored by ‘minitab’, ‘s’, ‘sas’, ‘spss’, ‘stata’, ‘systat’, ‘weka’, ‘dBase’.

[27] Wickham H. Ggplot2: elegant graphics for data analysis. New York: Springer-Verlag; 2016. Retrieved from https://cloud.r-project.org/web/packages/ggplot2/index.html

[28] Wickham H (2017). Tidyverse: Easily install and load the ‘tidyverse’.

[29] Wickham H, Hester J, Chang W (2018). Devtools: Tools to make developing r packages easier.

[30] Houben M, Noortgate WVD, Kuppens P (2015). The relation between short-term emotion dynamics and psychological well-being: a meta-analysis. Psychol Bull.

[31] Jahng S, Wood PK, Trull T (2008). Analysis of affective instability in ecological momentary assessment: indices using successive difference and group comparison via multilevel modeling. Psychol Methods.

[32] Wu H, Mata J, Furman DJ, Whitmer AJ, Gotlib IH, Thompson RJ. Anticipatory and consummatory pleasure and displeasure in major depressive disorder: An experience sampling study. Journal of abnormal psychology. 2017;126(2):149. 10.1037/abn0000244 PMC5305427 27936838

[33] Wichers M, Peeters F, Rutten BP, Jacobs N, Derom C, Thiery E (2012). A time-lagged momentary assessment study on daily life physical activity and affect. Health Psychol.

[34] Gjessing H, Aalen O, Borgan. Survival and event history analysis: A process point of view. New York: Springer Verlag; 2008.

[35] Thereau T, Grambsch P (2001). Modeling survival data: extending the cox model (statistics for biology and health).

[36] Mills M. Introducing survival and event history analysis: Sage; 2010.

[37] Van Roekel E, Vrijen C, Heininga VE, Masselink M, Bos EH, Oldehinkel AJ (2017). An exploratory randomized controlled trial of personalized lifestyle advice and tandem skydives as a means to reduce anhedonia. Behav Ther.

[38] Wichers MC, Myin‐Germeys I, Jacobs N, Peeters F, Kenis G, Derom C, Vlietinck R, Delespaul P, Van Os J (2007). Evidence that moment‐to‐moment variation in positive emotions buffer genetic risk for depression: a momentary assessment twin study. Acta Psychiatr Scand.

[39] Li J, Ji L (2005). Adjusting multiple testing in multilocus analyses using the eigenvalues of a correlation matrix. Heredity.

