# Supplementary material for: The dynamical signature of anhedonia in major depressive disorder: positive emotion dynamics, reactivity, and recovery
Source: BMC Psychiatry. 2019 Feb 8;19:59. doi: 10.1186/s12888-018-1983-5 (PMC6368777; doi:10.1186/s12888-018-1983-5)
Supplement: Supplementary file 1 — Anhedonia in MDD – supplementary analyses.pdf. The dynamical signature of anhedonia in Major Depressive Disorder: Positive emotion dynamics, reactivity, and recovery’. The datasets used for these analyses are: 1) MyData supplement agg Heininga 2018 07 26_ANONYM.sav, and 2) MyData supplement Heininga 2018 07 26_ANONYM.sav. These Additional file 1 include: Statistical procedures, including deviations from what was preregistered (https://osf.io/4bkad/). (PDF 665 kb) [file 12888_2018_1983_MOESM1_ESM.pdf]

Supplementary materials belonging to: ‘The dynamical signature of anhedonia in Major Depressive Disorder: Positive emotion dynamics, reactivity, and recovery’

Vera E. Heininga[1], Egon Dejonckheere[1], Marlies Houben[1], Jasmien Obbels[2], Pascal Sienaert[2], Bart Leroy[2], Joris van Roy[3], & Peter Kuppens[1]

[1] Research group of Quantitative Psychology and Individual Differences, KU Leuven, Belgium

[2] KU Leuven, University Psychiatric Center KU Leuven, Academic Center for ECT and Neuromodulation, Leuven/Kortenberg, Belgium

[3] Psychiatric Hospital Alexianen Tienen, Belgium

Author Note

Correspondence concerning this article should be addressed to Vera E. Heininga, Tiensestraat 102 - bus 3713, 3000 Leuven. E-mail: vera.heininga@kuleuven.be. Email addresses of all other authors are respectively: egon.dejonckheere@kuleuven.be; marlies.houben@kuleuven.be; jasmien.obbels@upckuleuven.be; pascal.sienaert@upckuleuven.be; bart.leroy@upckuleuven.be; joris.van.roy@fracarita.org; peter.kuppens@kuleuven.be

Abstract

These supplementary materials include: Statistical procedures, including deviations from what was preregistered (https://osf.io/gmfsc/); Supplementary exploratory analyses to H7a: Robustness of PA recovery slope model; preregistered follow up analyses when controlling for MDD severity; Exploratory analyses in which we explore whether our results would have been different if we would have excluded the ‘euphoria’-item from our PA scale, and, finally; preregistered exploratory analyses to validate a newly developed Ecological Momentary Aassessment (EMA) item of consummatory anhedonia. Preregistration doi:10.17605/osf.io/gjaze; Data and Materials:osf.io/8gxrw; Preprint doi:10.31234/osf.io/cfkts.

Keywords: Depression; Consummatory anhedonia; Experience Sampling Method (ESM); Daily life; Positive emotions; Positive affect; Emotion dynamics; Pleasure loss; Reward; Mood brightening effect

Supplementary materials belonging to: 'The dynamical signature of anhedonia in Major Depressive Disorder: Positive emotion dynamics, reactivity, and recovery'

Methods

Descriptives

Kurtosis and skewness. All variables were normally distributed, except age and compliance. Age showed a right-tailed more flat distribution than normal (kurtosis = 1.94; skewness = 0.35). Compliance rates showed a left-tailed more peaked distribution than normal (kurtosis = 4.29; skewness = -1.24). Hence, we calculated medians of these variables. The median age was 35 (31.50 in the control group, and 41 in the MDD anhedonia group). The median compliance rate was 91.43% (93.57% in the control group, and 87.14% in the MDD anhedonia group).

Statistical procedures

All statistical confirmatory analyses and follow up analyses, as well as some of the exploratory analyses, were registered prior to accessing the data. The preregistration form, data, and manuscript can be downloaded via https://osf.io/gmfsc/.

H1. To investigate whether anhedonia in MDD is associated with a less frequent reward experience, we estimated a multilevel logistic regression model with the frequency of Psychological Reward and Behavioral Reward being predicted by a random intercept by group (i.e., controls or MDD anhedonia)[1]:

[1] Note: The use of the natural logarithm was deemed most appropriate here, but deviates from what was preregistered

Level 1 (Equation 1a):

$$ln(\frac{p(Reward_{ij})}{1-p(Reward_{ij})})\ or\ ln(\frac{p(BR_{ij})}{1-p(BR_{ij})}) = \beta_{0j} + e_{ij}$$

Level 2 (Equation 1b):

$$\beta_{0j} = \gamma_{00} + \gamma_{01}(Group_j) + u_{0j}$$

H2. To investigate whether anhedonia in MDD is associated with a lower level of PA, we tested whether the random intercepts of PA differed between groups (Equation 2b):

Level 1 (Equation 2a):

$$PA_{ij} = \beta_{0j} + e_{ij}$$

Level 2 (Equation 2b):

$$\beta_{0j} = \gamma_{00} + \gamma_{01}(Group_j) + u_{0j}$$

H3. To investigate whether anhedonia in MDD is associated with more variability in PA, we tested whether the average within-person PA variance differed between the two groups in a one-sided Welch Two Sample t-test.

H4. To investigate whether anhedonia in MDD is associated with more instability in PA, we tested whether the Root Mean Successive Differences differed by group (Jahng, Wood & Trull, 2008; Equation 3b)[2]:

Level 1 (Equation 3a):

$$ln(SSD+1)_{ij} = \beta_{0j} + e_{ij}$$

[2] Note: This deviated from what was preregistered. According to Equation 15-17 in Jahng, Wood & Trull (2008), the SSD can be modeled by a generalized multilevel model with a gamma error distribution and log link (i.e., $log((PA_{i+1j} - PA_{ij})^2)_{ij} = \beta_{0j} + e_{ij}$).

Level 2 (Equation 3b):

$$\beta_{0j} = \gamma_{00} + \gamma_{01}(Group_j) + u_{0j}$$

H5. To investigate whether MDD anhedonia is associated with more inert PA, we tested whether the groups differed in their autocorrelation of person-mean centered PA (Equation 4c; $\gamma_{11}(Group_j)$):

Level 1 (Equation 4a):

$$PA_{ij} = \beta_{0j} + \beta_{1j}(PA_{i-1,j}) + \beta_{2j}(Reward_{ij})$$

Level 2 (Equation 4b-d):

$$\beta_{0j} = \gamma_{00} + \gamma_{01} + u_{0j}$$

$$\beta_{1j} = \gamma_{10} + \gamma_{11}(Group_j) + u_{1j}$$

$$\beta_{2j} = \gamma_{20} + \gamma_{21}(Group_j) + u_{2j}$$

H6. Simultaneously, to investigate whether MDD anhedonia is associated with a different PA reaction to rewards, we tested whether the groups differed in the level of PA at i after a reward between *i-1* and i had taken place (Equation 4d; $\gamma_{21}(Group_j)$), while controlling for person-mean centered PA at *i-1.* In case PA inertia did not differ by group, we reran the analyses while still controlling for the level of PA at *i-1* but after omitting its non-significant cross-level interaction (i.e., Equation 5c):

Level 1 (Equation 5a):

$$PA_{ij} = \beta_{0j} + \beta_{1j}(PA_{i-1,j}) + \beta_{2j}(Reward_{ij})$$

Level 2 (Equation 5b-d):

$$\beta_{0j} = \gamma_{00} + \gamma_{01} + u_{0j}$$

$$\beta_{1j} = \gamma_{10} + \gamma_{11} + u_{1j}$$

$$\beta_{2j} = \gamma_{20} + \gamma_{21}(Group_j) + u_{2j}$$

H7. To investigate whether anhedonia in MDD is associated with a faster decrease or recovery in PA after the initial increase in PA in reaction to a reward, we tested for group differences in PA recovery slope and duration.

Slope.

To investigate whether anhedonia in MDD is associated with a steeper slope, we tested whether the groups differed in their difference score of PA (i.e., $PA_{i+1j} - PA_{ij}$[3]) after a reward (Equation 6d), while controlling for differences in person-mean centered PA reactivity to reward (operationalized here as $PA_{ij} - PA_{i-1j}$; Equation 6b) as well as controlling for differences in minutes between *i* and *i+1* ("Time" variable; Equation 6c):

Level 1 (Equation 6a):

$$(PA_{i+1j} - PA_{ij}) = \beta_{0j} + \beta_{1j}(PA_{ij} - PA_{i-1j}) + \beta_{2j}(Time) + \beta_{3j}(Reward_{ij}) + e_{ij}$$

Level 2 (Equation 6b-d):

$$\beta_{0j} = \gamma_{00} + \gamma_{01} + u_{0j}$$

$$\beta_{1j} = \gamma_{10} + \gamma_{11} + u_{1j}$$

$$\beta_{2j} = \gamma_{20} + \gamma_{21} + u_{2j}$$

$$\beta_{3j} = \gamma_{30} + \gamma_{31}(Group_j) + u_{3j}$$

[3] $PA_{i+1j} - PA_{ij}$ was only calculated if no (additional) reward was reported on *i+1*

Duration. To investigate whether the time needed for PA recovery after rewards is shorter in MDD patients with anhedonia than in healthy controls, we tested whether the groups differed in the number of minutes participants needed to come back to baseline after a reward (i.e., the number minutes after *i* up until PA was equal or below the level of PA at *i-1*, the level before the reward was encountered) while controlling for the person-mean centered PA reactivity to reward (i.e., $(PA_{ij} - PA_{i-1,j})$) in the following random intercepts model[4]:

Level 1 (Equation 6a):

$$Recovery\, in\, minutes_{ij} = \beta_{0j} + \beta_{1j}(PA_{ij} - PA_{i-1,j}) + e_{ij}$$

Level 2 (Equation 6b-d):

$$\beta_{0j} = \gamma_{00} + \gamma_{01}(Group_j) + u_{0j}$$

$$\beta_{1j} = \gamma_{10} + u_{2j}$$

Familywise error rate control

In total, we tested 12 different but comparable models of PA reactivity (namely reactivity to psychological rewards and behavioral rewards; in a full and a trimmed model; followed by rerunning these four tests in the exploratory analyses with regard to PA2 and the newly developed ESM-item).

In order to maintain a familywise error rate of .05, we applied a VeffLi-Bonferroni-correction to the models of PA reactivity and PA recovery. The effective number of independent PA reactivity interactions were 9, making the significance threshold

[4] It should be noted that we restricted the number of assessments in which the recovery could take place to *i+5* (i.e., on average six hours after the reward was reported), and that we only calculated the number of minutes if no "new" reward was reported before PA was recovered. For the exact computations, please see our programming code in SPSS language in the supplementary material called "SPSS programming syntax".

that is required to keep Type I Error Rate at 5% with 9 independent variables $1 - (1 - 0.05)^{(1/9)} = 0.01$.

With regard to PA recovery, we also tested 14 different models in total, namely: recovery after psychological rewards and behavioral rewards; with two alternative ways of modelling PA recovery; followed by rerunning the first two in the exploratory analyses with regard to PA2 and the newly developed ESM-item; complemented by two tests of the PA recovery duration after psychological rewards and behavioral rewards; and re-iterated for PA2. The effective number of independent PA reactivity interactions were 10, making the significance threshold that is required to keep Type I Error Rate at 5% with 10 independent variables $1 - (1 - 0.05)^{(1/10)} = 0.01$.

Supplementary material to H7a: Robustness of PA recovery slope model

We preregistrated that we would operationalized the PA Recovery slope as "$PA_{i+1} - PA_i | PE_i$, meaning that, if a Psychological Reward took place, a difference score is calculated between the first and second assessment of PA thereafter. To control for differences in participants' initial increase in PA (i.e., PA reactivity to PE, measured at assessment i), PA reactivity was operationalized as $PA_i - PA_{i-1} | Reward_i$, person-mean centered, and included as a covariate. In addition, to control for the different duration of assessment intervals, the time passed between assessments was used as a covariate." The time passed was measured as the number of minutes between *t* and *t+1*.

In addition to the model that we preregistrated, we came up with two additional models of how we could have modeled the PA recovery slope model to test our hypothesis that the PA recovery slope was steeper in individuals with MDD and anhedonia than in those individuals without such diagnoses.

To investigate whether the PA recovery slope decreases more steeply in MDD patients

with anhedonia versus controls after reward experiences ($p < .05$), we controlled for the person-mean centered level of PA at time point i, the time point right after a reward was experienced (Reward or BR), PA_{i+1j} reflects the amount of decrease in PA on one assessment after the initial increase in PA in reaction to experiencing a reward. Again, the slopes were separately modelled with regard to Psychological Rewards and Behavioral Rewards, that were reported on i (and thus experienced somewhere between i and *i-1*):

Level 1 (Equation 4a):

$$PA_{i+1j} = \beta_{0j} + \beta_{1j}(PA_{ij}) + \beta_{2j}(Reward_{ij}) + e_{ij}$$

Level 2 (Equation 4b-d):

$$\beta_{0j} = \gamma_{00} + \gamma_{01}(Anhedonia_j) + u_{0j}$$

$$\beta_{1j} = \gamma_{10} + \gamma_{11}(Anhedonia_j) + u_{1j}$$

$$\beta_{2j} = \gamma_{20} + \gamma_{21}(Anhedonia_j) + u_{2j}$$

As shown in Supplementary STable 1, the first alternative option showed that, while controlling for PA at t, the experience of a Psychological Reward orBehavioral Reward between t and t-1 is unrelated to the level of PA on t+1 . Similarly, inalternative 2, while controlling for the person-mean centered initial increase in PA immediately thereafter (i.e., on t), the experience of a Psychological Reward between t and t-1 is unrelated to the level of PA on t+1. It should be noted, however, that these relationship coeficients are conditional on the value of Anhedonia. The coefficient for Psychological Reward reflects the effect of Psychological Reward in the control group (i.e., when Anhedonia=0); whereas the coefficient for anhedonia is the effect of anhedonia when there is no reward experienced (i.e., when PE=0).

As shown in Supplementary STable 2, the second alternative option showed that, while controlling for the person-mean centered initial increase in PA immediately thereafter on t,

the experience of a Behavioral Reward between t and t-1 is linked to an increased level of PA on t+1 in the control group. Nevertheless, there is no difference in the effect of Behavioral Reward between patients with MDD and anhedonia and healthy controls.

Follow up analyses

Controlling for MDD severity

In a meta-analysis, the effect sizes of emotional reactivity in MDD showed considerable heterogeneity (Bylsma et al., 2008). In his recent review on emotions in depression, Rottenberg (2017) points towards severity or persistency of the depressive episodes as possible moderators for the counterintuitive Mood brightening effects found in the ESM studies.

We registered prior to accessing the data to investigate whether to control for depression severity as measured after the ESM study (see: https://osf.io/gmfsc/). However, in hindsight, this data was not available and we used what was available: Depression severity before the ESM study.

The level of depression severity was measured with the Quick Inventory of Depressive Symptomatology Self Report (QIDS-16-SR; Rush et al., 1996). After the introductory text "please checkmark the one response to each item that is most appropriate to how you have been feeling over the past 7 days", 16 statements followed on possible depressive states. Answer categories on each item range from 0 to 3. To get the total score, symptom domains are summed. For domains that require more than one item, the highest score of the item relevant for each domain is taken. For example, if early insomnia is 0, middle insomnia is 1, late insomnia is 3, and hypersomnia is 0, the sleep disturbance domain is rated 3. The total score ranges from 0-27, and can be categorized as: 1-5 = No depression; 6-10 = Mild

depression; 11-15 = Moderate depression; 16-20 = Severe depression; 21-27 = Very severe depression.

Depression severity as measured by the QIDS was close to a normal bell shape, and only very slightly skewed to the left with a skewness value of 0.17 and kurtosis value of 1.50. In line with the grouping according to our selection criteria, the groups differed significantly in their depression severity ($\Delta M = -14.10$, 95% CI $[-14.29, -13.91]$, $t(5, 112.55) = -147.17$, $p < .001$). As shown in STable 3, participants in the control group had an average QIDS sum score that could be categorized as no depression whereas participants in the MDD anhedonia group had an average QIDS sum score that could be categorized as a severe depression.

Although we preregistrated to do follow-up analyses with depression severity as covariate, depression severity was found to highly correlated to anhedonia ($r = .87$, 95% CI [.81, .92], $t(85) = 16.61$, $p < .001$). Because multicollinearity causes the coefficient estimates to behave erratically in response to small changes in the model, in hindsight, and different from what we preregistrated, we decided to ommit these follow-up analyses.

Exploratory analyses

Influence of "Euphoria"

These exploratory analyses were not preregistered, and became of interest after accessing the data, plotting the data, and calculating its cronbachs alpha. Distribution plots of the PA items showed that feeling happy and relaxed where normally distributed, whereas feeling euphoric was heavily skewed towards zero (see SFigure 1). Cronbachs alpha improved from .03 to .44 when leaving out the euphoria item.

To investigate the impact of the "Euphoria"-item on our results, we reran the relevant analyses while omitting the scores on the euphoria item and thus using the average PA

scores of feeling happy and feeling relaxed. Cronbach's alpha reliability was .44 within-subject, and .99 between subject.

- Similar to the original results of H2, compared to controls, MDD patients with anhedonia reported a less level of PA2 ($t(84.96)$ = -7.72, $p < 0.001$).
- Similar to the original results of H3, the variance in PA2 was not statistically significant $\Delta M = 16.17$, 95% CI $[-49.31, 81.64]$, $t(79.87) = 0.49$, $p = .624$.
- Similar to the original results of H4, there was no statistically significant difference in PA2 instability between controls and MDD patients with anhedonia (B = -0.18; $t(85.42)$ = -1.24; p = 0.110).
- Similar to the original results of H5, MDD patients with anhedonia did not have a stronger autocorrelation of PA2 and thus not more inert PA2 (B = -0.01; $t(4349)$ = -0.49; p = 0.312).
- Similar to the original results of H6, MDD patients with anhedonia showed a stronger PA2 reactivity to Psychological Rewards (B = 3.65; $t(57)$ = 2.53; p = 0.014), but not to Behavioral Rewards (B = -1.50; $t(76)$ = -1.44; p = 0.155).
- Similar to the original results of H7 on a steeper slope, MDD patients with anhedonia did not show a faster PA2 recovery after a Psychological Rewards (B = -1.63; $t(68)$ = -1.10; p = 0.137), nor after a Behavioral Rewards (B = 0.33; $t(78)$ = 0.23; p = 0.410).
- Similar to the original results of H7 on a shorter duration, the average minutes to return to baseline (i.e., the level of PA2 at i-1) after experiencing Psychological Rewards were 87.93, , and for Behavioral Rewards 96.66. Patients with MDD and anhedonia did not show a faster PA2 recovery after Psychological Rewards (B = -4.27; $t(262)$ = -0.65; p = 0.259), nor after Behavioral Rewards (B = 8.19; $t(245)$ = 1.03; p = 0.152).

In conclusion, results were all in the same direction when using PA calculated as a mean of happy and relaxed (instead of happy, relaxed, and euphoric).

Validation of newly developed ESM-item

These exploratory analyses were registered prior to accessing the data (see: https://osf.io/gmfsc/). To validate our newly developed self-reported ESM measure of consummatory anhedonia, we explore the properties of the momentary item: “To what degree do you find it difficult to experience pleasure in activities at the moment?”, with a sliding scale to answer somewhere between 0 anchored “not at all”, and 100 anchored with “very difficult”.

Descriptives. For a distribution of the anhedonia scores measured by ESM by group, please see SFigure 2. This SFigure depicts all datapoints (jittered horizontally), the central tendencies of the data (the vertical bar), with a rectangle representing the 95% confidence inference interval, and a smoothed density of the data (colored). In line with the central tendencies displayed visually, the results of the t-test showed that participants from the MDD anhedonia group reported significantly more momentary anhedonia than controls ($\Delta M = -46.48$, 95% CI $[-47.32, -45.64]$, $t(5,885.56) = -108.40$, $p < .001$).

Agreement between SCID, QIDS, and ESM-item. Next, we compared the agreement between our newly developed momentary ESM-item of anhedonia, and our two other measures of anhedonia: 1) anhedonia measured in retrospect by a trained clinian using the semi-structured interview SCID-I, and 2) the in retrospect self-reported anhedonia item measured by the QIDS-13.

As also described in the main article, anhedonia was assessed by the SCID-I question “Did you lose interest or pleasure in things you usually enjoyed? (What was that like?)” and, if yes, “When was that? Was that nearly every day? How long did it last? As long as two

weeks?". Based on the participants' answers during the assessment of the SCID-I, a researcher who was a trained clinician rated anhedonia as "absent" "subthreshold", or "present".

For the 13th item of the QIDS questionnaire on general interest, participants could indicate (0) My normal interest in other people or activities has not changed; (1) I notice that I have less interest in other people or activities; (2) I am only interested in one or two of the activities I used to have; (3) I have practically no interest in activities that I used to have.

These exploratory analyses were registered prior to accessing the data (see: https://osf.io/gmfsc/). Because the QIDS was only assessed before ESM study and not thereafter, please note that we substituted step 2 by a different correlation than originally preregistrated. In addition, we used a slightly different statistical test than we preregistered.

Step 1: SCID VS QIDS-13.

The correlation between the patients' self-reported 13th item of the QIDS of loss of interest over the last two weeks, and the clinian-rated measure of loss of interest or pleasure in things the patient usually enjoyed over the last two weeks was $r = .65$, 95% CI [.51, .76], $t(85) = 7.86$, $p < .001$.

Step 2: SCID VS ESM-item.

The correlation between the clinian-rated measure of loss of interest or pleasure in things the patient usually enjoyed over the last two weeks, and the median of patients' degree to which they found it difficult to experience pleasure in activities at ten semi-random momentary assessment in the two weeks that followed after baseline SCID diagnosis was $r = .80$, 95% CI [.71, .87], $t(85) = 12.46$, $p < .001$.

Step 3: QIDS-13 VS ESM-item of momentary anhedonia.

The correlation between the patients' self-reported 13th item of the QIDS of loss of interest over the last two weeks, and the median of patients' degree to which they found it difficult to experience pleasure in activities at ten semi-random momentary assessment in the two weeks that followed after baseline SCID diagnosis was $r = .53$, 95% CI [.37, .67], $t(85) = 5.83$, $p < .001$.

Difference in results when using a continuous and momentary instead of a categorical and retrospective measure of anhedonia. We reran the analyses using anhedonia as measured by the median of the momentary ESM-item, and found the following:

H1: Less rewards

Logistic regressions indicated that the level of median momentary MDD anhedonia was not related to a lower frequency of Psychological Rewards (B = -0.01; z = -1.37; p = 0.086). The results also indicated that the higher a participant's median anhedonia, the lower the frequency of Behavioral Rewards (B = -0.01; z = -2.09; p = 0.018). The effect size of the latter was negligible, and therefore of low practical significance. ## H2: Lower level of PA

Controls reported an average level of PA of 46.55 (*t(84.81)* = 23.73; $p < 0.001$), and for each point that participants' median anhedonia was lower, their PA was -0.33 lower (*t(84.91)* = -8.02, $p < 0.001$).

H3: More variability in PA

The variance in PA was not statistically significantly linked to the grouping factor ($r = -.11$, 95% CI $[-.32, .10]$, $t(85) = -1.06$, $p = .293$).

H4: More instability in PA

There was not more instability in those participants with more severe levels of anhedonia ($B = 0$; *t(83.37)* = -1.63; $p = 0.053$).

H5: More inert PA

Higher levels of median anhedonia were not related to more inert PA ($B = 0$; *t(4369)* = -0.95; $p = 0.170$).

H6: Different PA reactivity to rewards

Momentary median anhedonia was related to differences in PA reactivity, with one unit more severe anhedonia linked to a 0.07 increase in PA after experiencing Psychological Rewards ($B = 0.07$; *t(4419)* = 3.87; $p = < 0.001$).

H7: Faster PA recovery

Steeper slope. Median momentary anhedonia was not related to a faster recovery of PA after Psychological Rewards ($B = -0.04$; *t(3086)* = -1.98; $p = 0.024$), nor after Behavioral Rewards ($B = -0.01$; *t(2803)* = -0.52; $p = 0.300$)

Shorter duration. Median momentary anhedonia was not related to less minutes needed to return to baseline after Psychological Rewards ($B = -0.08$; *t(262)* = -0.70; $p = 0.241$), nor after Behavioral Rewards ($B = 0.11$; *t(245)* = 0.83; $p = 0.203$).

References

Comtois, D. (2018). *Summarytools: Tools to quickly and neatly summarize data.* Retrieved from https://CRAN.R-project.org/package=summarytools

Elff, M. (2017). *Memisc: Management of survey data and presentation of analysis results.* Retrieved from https://CRAN.R-project.org/package=memisc

Komsta, L., & Novomestky, F. (2015). *Moments: Moments, cumulants, skewness, kurtosis and related tests.* Retrieved from https://CRAN.R-project.org/package=moments

Kuznetsova, A., Brockhoff, P. B., & Christensen, R. H. B. (2017). lmerTest package: Tests in linear mixed effects models. *Journal of Statistical Software*, *82*(13), 1–26. doi:10.18637/jss.v082.i13

Lüdecke, D. (2018). *SjPlot: Data visualization for statistics in social science.* doi:10.5281/zenodo.1308157

Patil, I. (2018). *Ggstatsplot: 'Ggplot2' based plots with statistical details.* Retrieved from https://CRAN.R-project.org/package=ggstatsplot

Phillips, N. (2017). *Yarrr: A companion to the e-book "yarrr!: The pirate's guide to r".* Retrieved from https://CRAN.R-project.org/package=yarrr

R Core Team. (2017). *Foreign: Read data stored by 'minitab', 's', 'sas', 'spss', 'stata', 'systat', 'weka', 'dBase', ...* Retrieved from https://CRAN.R-project.org/package=foreign

Wickham, H. (2016). *Ggplot2: Elegant graphics for data analysis.* Springer-Verlag New York. Retrieved from http://ggplot2.org

Wickham, H. (2017). *Tidyverse: Easily install and load the 'tidyverse'.* Retrieved from

https://CRAN.R-project.org/package=tidyverse

Wickham, H., Hester, J., & Chang, W. (2018). *Devtools: Tools to make developing r packages easier*. Retrieved from https://CRAN.R-project.org/package=devtools

Table 1

Recovery slope from PA reactivity to rewards: alternative 1

	Psychological Reward					Behavioral Reward				
	Estimate	Std. Error	df	t value	Pr(>|t|)	Estimate	Std. Error	df	t value	Pr(>|t|)
(Intercept)	42.79	1.74	86.00	24.56	0.00	43.57	1.78	85.40	24.52	0.00
Reward	-0.07	0.70	51.59	-0.09	0.93	0.45	0.65	81.21	0.69	0.49
Anhedonia	-19.15	2.36	85.12	-8.10	0.00	-18.97	2.41	84.90	-7.86	0.00
PA_c	0.29	0.01	4,728.26	21.04	0.00	0.30	0.01	4,707.95	21.96	0.00
Reward_plus_1	7.79	0.44	4,801.23	17.64	0.00	2.78	0.41	4,685.66	6.71	0.00
Time2	0.00	0.00	4,786.50	4.43	0.00	0.00	0.00	4,782.57	4.18	0.00
Reward:Anhedonia	1.43	0.97	58.24	1.47	0.15	0.54	0.88	71.52	0.61	0.54

Note. Dependent variable is Positive Affect (PA) on time point t+1; PA is the average of feeling relaxed, happy, and euphoric; Time is the time past since the last assessment in minutes; laggedPA is the person-mean centered lagged variable of PA (i.e., PA on t-1); PAreactivity is the person-mean centered amount of increase in PA on time point t in comparison to t-1 (i.e., PA reactivity, but now modelled as a difference score: PA minus PA on t-1); Reward refers to positive event experienced somewhere between t-1 and t.

Table 2

Recovery slope from PA reactivity to rewards: alternative 2

	Psychological Reward					Behavioral Reward				
	Estimate	Std. Error	df	t value	Pr(>|t|)	Estimate	Std. Error	df	t value	Pr(>|t|)
(Intercept)	42.40	1.73	86.22	24.54	0.00	42.02	1.72	85.53	24.42	0.00
Reward	1.04	0.85	41.57	1.23	0.23	1.85	0.79	72.04	2.34	0.02
Anhedonia	-19.76	2.34	85.25	-8.43	0.00	-18.83	2.34	84.82	-8.06	0.00
PAreactivity	0.04	0.01	3,960.16	2.96	0.00	0.05	0.01	3,944.19	3.78	0.00
Reward__plus__1	8.26	0.51	3,984.86	16.35	0.00	8.41	0.50	4,009.58	16.69	0.00
Time2	0.00	0.00	3,954.15	3.58	0.00	0.00	0.00	3,943.65	3.94	0.00
Reward:Anhedonia	3.82	1.19	47.21	3.20	0.00	-0.09	1.12	76.23	-0.08	0.93

Note. Dependent variable is Positive Affect (PA) on time point t+1; PA is the average of feeling relaxed, happy, and euphoric; Time is the time past since the last assessment in minutes; laggedPA is the person-mean centered lagged variable of PA (i.e., PA on t-1); PAreactivity is the person-mean centered amount of increase in PA on time point t in comparison to t-1 (i.e., PA reactivity, but now modelled as a difference score: PA minus PA on t-1); Reward refers to having experienced a positive event experienced somewhere between t-1 and t.

Table 3

Relevant variables in control groups and anhedonia MDD group.

	Control group				Anhedonia MDD group			
	Mean	SD	Min	Max	Mean	SD	Min	Max
QIDS_SUM	3.33	2.53	0.00	10.00	17.43	4.84	5.00	24.00
QIDS_13	0.00	0.00	0.00	0.00	1.02	0.82	0.00	3.00
Anhedonia_ESM_med	12.61	13.92	0.00	63.00	59.10	19.77	17.00	100.00
PA	44.17	11.58	19.96	67.75	25.43	11.04	5.34	58.74
PA2	53.56	10.75	26.66	77.91	30.57	11.79	8.01	61.01
PA_var	161.36	116.41	39.68	584.36	143.09	108.37	37.36	483.11
PA2_var	235.82	159.16	47.54	750.10	219.65	145.29	42.70	636.11
Relaxed	56.29	10.22	37.86	81.23	33.90	11.67	10.18	59.92
Happy	50.83	13.77	12.14	74.59	27.24	13.50	2.58	62.61
Euphoric	25.38	19.28	0.16	65.34	15.15	12.62	0.00	54.20

Note. 'QIDS_SUM' refers to the sumscore of depression as measured by the QIDS; 'QIDS_13' refers to the score on the anhedonia item as measured by the QIDS; 'Anhedonia_ESM_med' refers to the within-person median of the momentary ESM item across the whole study period; PA is the average of the EMA items feeling relaxed, happy, and euphoric.

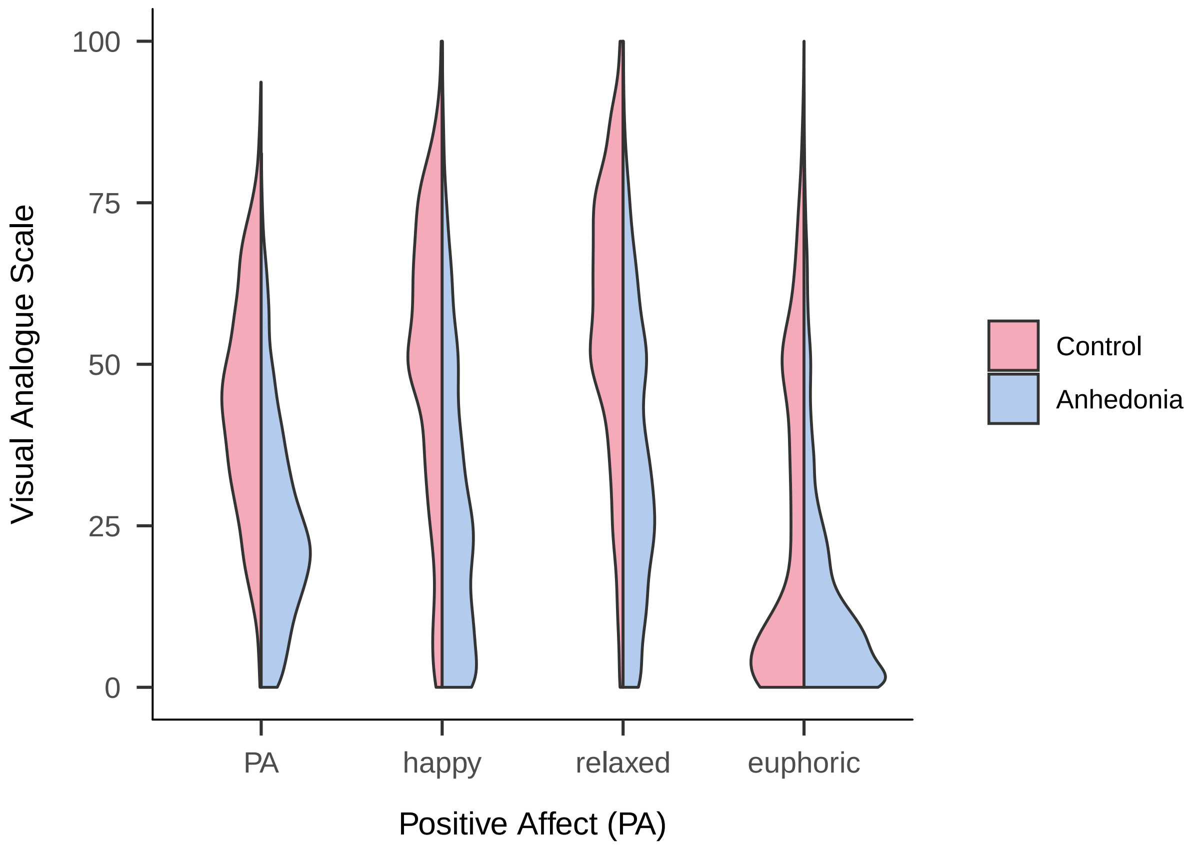

Figure 1. Distribution of Positive Affect (PA)

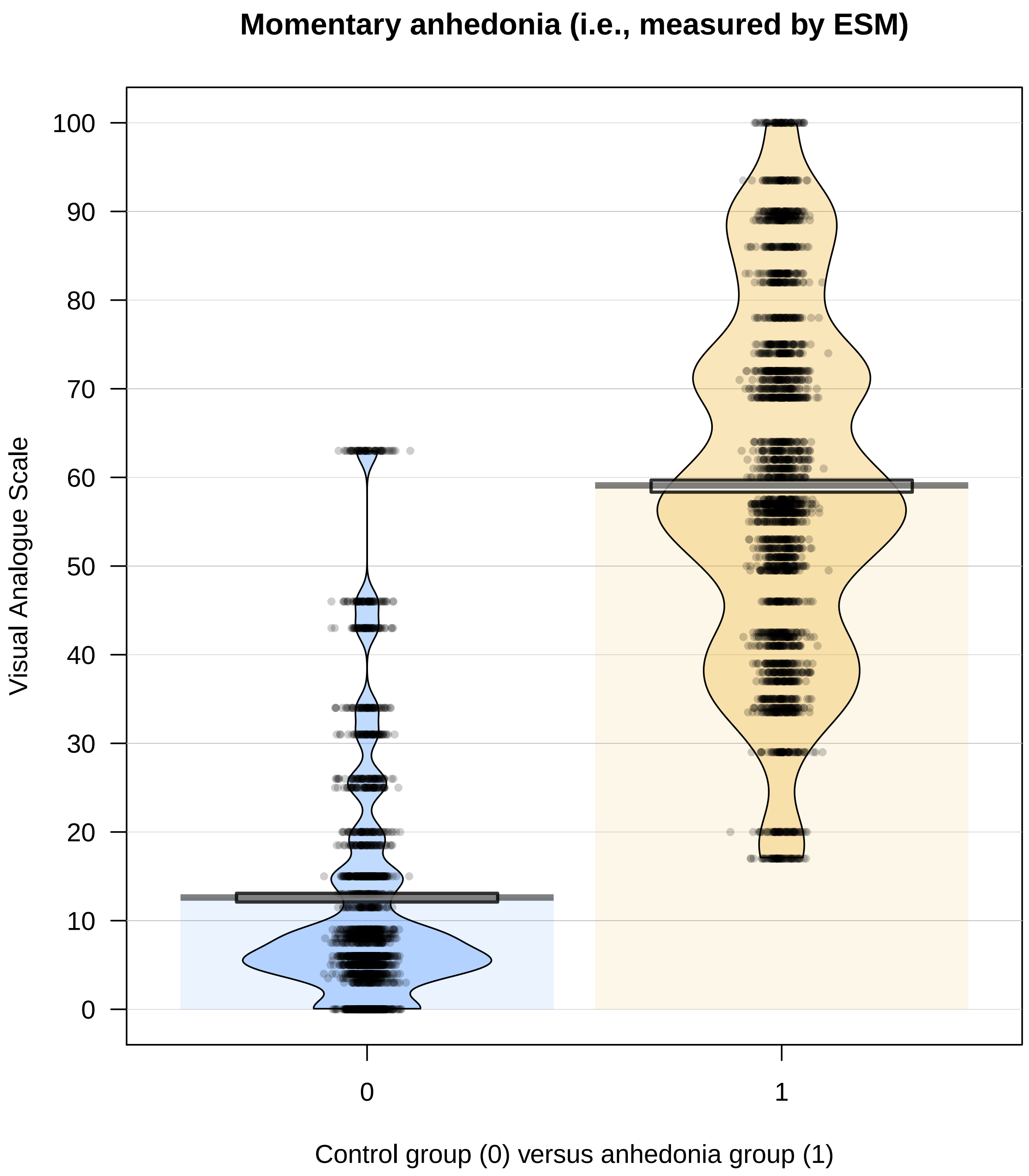

Figure 2. Descriptives of variables relevant to exploratory analyses
